# Long-Term Effects of Climate and Competition on Radial Growth, Recovery, and Resistance in Mongolian Pines

**DOI:** 10.3389/fpls.2021.729935

**Published:** 2021-09-14

**Authors:** ShouJia Sun, JinSong Zhang, Jia Zhou, ChongFan Guan, Shuai Lei, Ping Meng, ChangJun Yin

**Affiliations:** ^1^Key Laboratory of Tree Breeding and Cultivation of National Forestry and Grassland Administration, Research Institute of Forestry, Chinese Academy of Forestry, Beijing, China; ^2^Collaborative Innovation Center of Sustainable Forestry in Southern China, Nanjing Forestry University, Nanjing, China

**Keywords:** Mongolian pine, climate change, competition index, radial growth, vulnerability to drought

## Abstract

Understanding the response of tree growth and drought vulnerability to climate and competition is critical for managing plantation forests. We analyzed the growth of Mongolian pines in six forests planted by the Three-North Shelter Forest Program with tree-ring data and stand structures. A retroactive reconstruction method was used to depict the growth-competition relationships of Mongolian pines during the growth period and their climatic responses under different competition levels. Drought vulnerability was analyzed by measuring the basal area increment (BAI) of different competition indices (CIs). In young trees, differences in BAIs in stands with different CIs were not statistically significant. After 15–20 years, medium- and high-CI stands had significantly lower tree-ring widths (TWs) and BAIs than the low-CI stands (*p* < 0.05). The standardized precipitation evapotranspiration index (SPEI), precipitation, relative humidity, and vapor pressure deficit were major factors affecting tree growth. On a regional scale, climate outweighed competition in determining radial growth. The relative contribution of climatic factors increased with the gap in SPEI between plantation sites and the native range, while the reverse pattern of the competition-growth relationship was observed. Drought reduced TWs and BAIs at all sites. Stands of different CIs exhibited similar resistance, but, compared with low-CI stands, high- and medium-CI stands had significantly lower recovery, resilience, and relative resilience, indicating they were more susceptible to drought stresses. Modeled CI was significantly negatively related to resistance, resilience, and relative resilience, indicating a density-dependence of tree response to drought. After exposure to multiple sequential drought events, the relative resilience of high-CI stands decreased to almost zero; this failure to fully recover to pre-drought growth rates suggests increased mortality in the future. In contrast, low-CI stands are more likely to survive in hotter, more arid climates. These results provide a better understanding of the roles of competition and climate on the growth of Mongolian pines and offer a new perspective for investigating the density-dependent recovery and resilience of these forests.

## Introduction

Satellite data acquired by the National Aeronautics and Space Administration (NASA) of the USA indicate that the global forest area increased by 5.18 × 10^8^ ha in the past two decades, with major contributions from China and India. For China, 42% of this increase is attributed to afforestation programs (Chen et al., 2019). The Three-North Shelter Forest Program (TNSFP) is the largest afforestation project launched worldwide. Since its initiation in 1978, the program has planted 4.61 × 10^7^ ha of forests in North China. The Mongolian pine (*Pinus sylvestris* var. mongolica Litv.), native in the Hulunbuir Sandy Land and Great Khingan Mountains, is a major species selected for the TNSFP because of its tolerance to drought and cold stresses. Mongolian pines have been introduced to TNSFP shelterbelts in other regions because they are adaptable and are effective for windbreaks and sand fixation. They currently cover >3.0 × 10^6^ ha. However, in recent years, an extensive decline in Mongolian pines has been observed in TNSFP shelterbelts (Zheng et al., 2012). A survey of shelterbelts in Liaoning Province found that 65.27% of Mongolian pines were declining or dead (Wu et al., 2014). Climate studies predict that the eastern part of the TNSFP area is likely to experience continuing warming and aridification in the future, suggesting there will be further extensive tree mortality.

The Fifth Assessment Report from the UN Intergovernmental Panel on Climate Change (IPCC) estimates that, from 1880 to 2012, the average global temperature increased by ~0.85°C, and the trend was even more pronounced in high latitudes (IPCC, 2014). Continual global climate change is expected to increase the frequency and severity of extreme climatic events (e.g., droughts, high temperature, and heat waves) (Neumann et al., 2017; Zas et al., 2020; Pokhrel et al., 2021), leading to a loss of susceptible species (Redmond et al., 2018), a decrease of forest productivity (Trugman et al., 2018), and an escalation of plant mortality (Greenwood et al, 2017; Neumann et al., 2017; Colangelo et al., 2018). Future aridification is likely to expose trees to greater drought stresses, particularly for high-density forests (Helluy et al., 2020). Competition leads to differences in growth between neighbors (Foster et al., 2016; Calama et al., 2019) and regulates individual responses to drought (Helluy et al., 2020). Therefore, the interaction between climate and competition and their impact on tree growth have become a focus of recent ecological studies (Jiang et al., 2018; Trugman et al., 2018; Liang et al., 2019).

Under the influence of climate and competition, forest stability is likely to be determined by the tolerance of trees, their subsequent recovery, and their abilities to maintain functions during and after stressful events (Redmond et al., 2018; Zas et al., 2020). Increasing evidence has indicated that drought events produce long-term physiological disorders associated with hydraulic segmentation, depletion of carbon reserves, and canopy defoliation in trees (Galiano et al., 2011), and different populations may respond to the events *via* different strategies (i.e., high resistance or high recovery) (DeSoto et al., 2020; Zas et al., 2020). Research studies have expanded the understanding of the characteristic response of trees to repeated droughts (Bigler et al., 2007; Anderegg et al., 2020). Recent studies revealed that the response of a tree to climate, competition, and site condition is determined by its competing environment (Clark et al., 2016; Calama et al., 2019), and intensifying competition in a stand increases the vulnerability of the trees to drought stress (Primicia et al., 2015; Zeng et al., 2020). Although climate, competition, and their interactions are known to influence the sensitivity of trees to drought stress (Clark et al., 2016; Gomes Marques et al., 2018), disentangling their individual effects remains challenging (Vanhellemont et al., 2019).

The construction of TNSFP shelterbelts introduced Mongolian pines from their native range to other regions. Trees distributed near the boundaries of the habitat range are expected to be sensitive to climate and competition, potentially becoming a good source for investigating their response to the two variables. Sufficient permanent sample stands and continuous long-term records of radial tree growth are lacking for these populations. However, retrospective analysis of tree-ring data provides an alternative approach (Foster et al., 2016; Calama et al., 2019; Zeng et al., 2020). Earlier studies on Mongolian pines in TNSFP shelterbelts have primarily focused on the growth-climate relationship (Bao et al., 2012), a hydraulic failure because of increased drought (Li et al., 2020), and the impact of land utilization on stand decline (Zheng et al., 2012). However, little is understood regarding the response of Mongolian pines distributed near the boundary of the native range to climate and competition and, in particular, how competition affects post-drought recovery in stands of different densities. This has limited our ability to predict the growth, decline, and restoration of Mongolian pine forests under the changing global climate.

This study investigated Mongolian pines in the native range [i.e., Hulunbuir (HLB)] and those distributed near the edges of the plantation forest range. Some plantation forests, particularly high-density stands, have exhibited a trend of decline and mortality. Therefore, we hypothesized that (1) the radial growth of Mongolian pines was affected by both climate and competition; (2) climate is a key factor affecting the radial growth of Mongolian pines, but the competition cannot be ignored; and (3) competition affects the sensitivity of Mongolian pines to drought stress. We measured the radial growth patterns of Mongolian pines in stands of different densities and established tree-ring chronologies. Then, a retroactive reconstruction method was developed to analyze the growth-competition relationship. Basal area increments (BAIs) were also measured and used to reconstruct competition indices for stands of different densities. The results were expected to provide insights into the future trends for decline and mortality of Mongolian pines through quantitative characterization of their response to climate and competition and their density-dependent post-drought recovery. Such insights may offer a critical basis for the scientific management of the Mongolian pine shelterbelts established by the TNSFP.

## Materials and Methods

### Experimental Sites and Sample Collection

**Figure 1:**
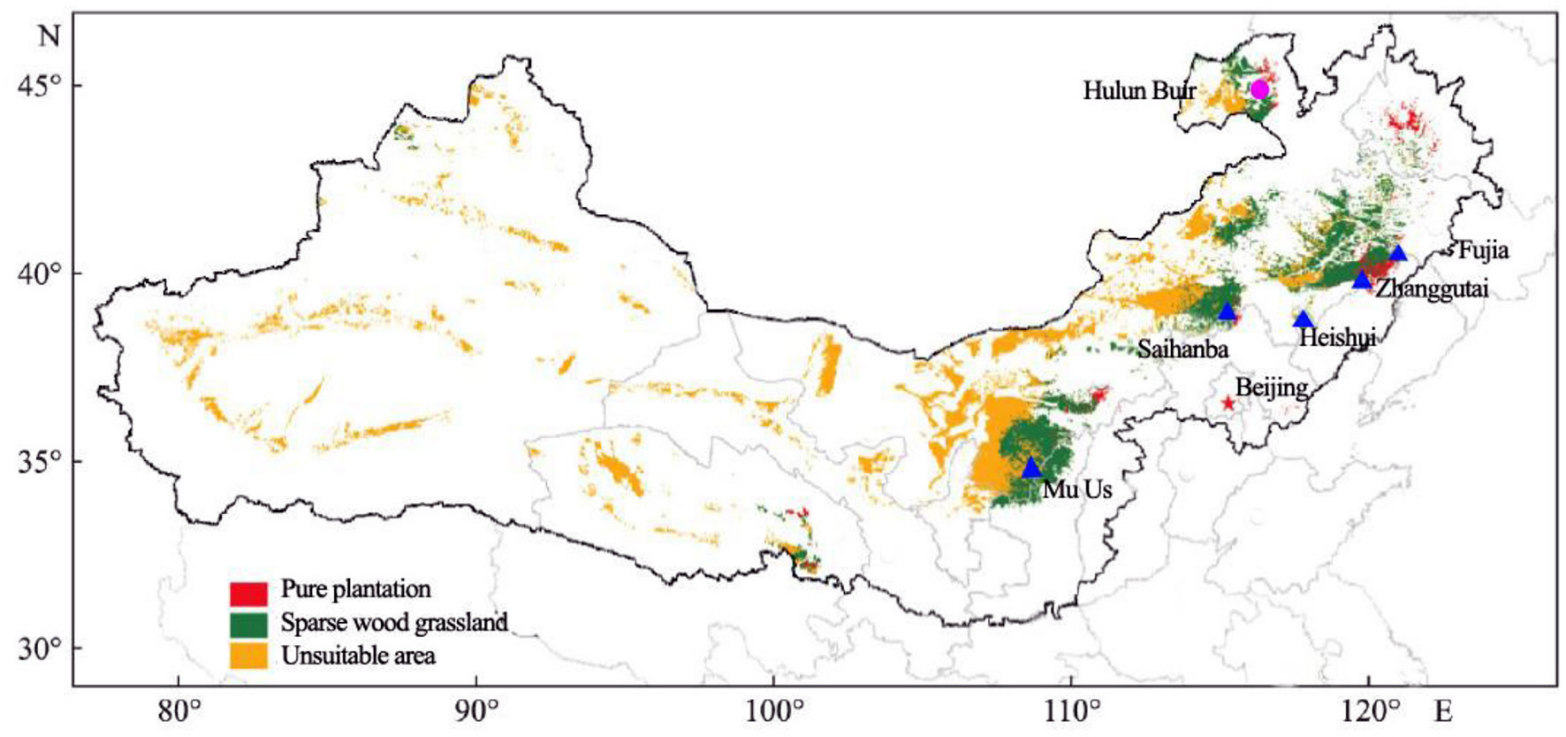
Experimental sites in the Three-North Shelter Forest. Blue triangles show sampled plantations of Mongolian pine. The pink circle shows a sample site of a natural forest.

One natural forest at Hulunbuir (HLB, Inner Mongolia, China) Sandy Land and five introduced plantation forests were selected for study (Figure 1). The plantation forests were located at Mu Us (MUS, Shanxi Province, China), Saihanba (SHB, Hebei Province, China), Heishui, Zhangutai, and Fujia (HEI, ZGT, FUJ: all Liaoning Province, China).

At all six sites, annual precipitations were <500 mm. Temperature and vapor pressure deficit (VPD) of SHB and HLB were obviously lower than those of the other sites, while SPEI was a reverse pattern (Table S1). Precipitation was predominantly distributed in July and August, and peak temperature occurred between June and August, showing a synchronization between precipitation and temperature (Figure S1). The MUS and HEI forest sites had high VPDs between May–June, reflecting dry summers and autumns. Sites at SHB and HLB had high SPEIs on a monthly scale, indicating relatively humid environments with low evapotranspiration rates. In contrast, ZGT showed a low SPEI on the monthly scale, corresponding to a more arid environment than the other sites.

At each sites, Mongolian pine plantations were established at different times with different densities (Table 1). Trees were selected from stands of the low, medium, and high densities at each site. Mongolian pines in low-density stands experienced low competition, with minimal crown overlap and excellent tree vigor. Medium-density stands showed moderate crown overlap and reasonably good vigor. High-density stands had severe crown overlap, needle loss at the middle-lower portion of the crown progressing upward, and evident signs of decline. Three 30 × 30 m sampling plots were set up in each stand, creating 54 plots in total. All the trees in each plot were measured for height, diameter at breast height (DBH), and geographical coordinates, allowing calculation of densities and competition indices (CIs). Site condition, namely site index, was defined as the height of the dominant trees at a stand age of 40 years, computed according to the model by An et al. (2019).

**Table 1:** The statistical description of low-competition index (L-CI), medium-competition index (M-CI), and high-competition index (H-CI) stands of Mongolian pine at six experimental sites in the Three-North Shelter Forest.

**Site**	**Type**	**Crown closure**	**Mean height (m)**	**Mean DBH (cm)**	**Stand density (n/ha)**	**Competition index**	**Established time**
MUS	L-CI	0.28 ± 0.07	12.8 ± 0.9	24.7 ± 2.1	214 ± 12	0.37 ± 0.08	1981–1985
	M-CI	0.58 ± 0.11	13.7 ± 1.1	19.3 ± 1.2	372 ± 31	1.78 ± 0.15	
	H-CI	0.89 ± 0.15	14.6 ± 1.2	17.6 ± 1.3	736 ± 48	3.43 ± 0.64	
SHB	L-CI	0.23 ± 0.06	16.1 ± 1.0	31.6 ± 2.1	252 ± 15	0.47 ± 0.10	1974–1978
	M-CI	0.45 ± 0.09	18.3 ± 1.4	26.7 ± 1.7	525 ± 27	1.84 ± 0.17	
	H-CI	0.85 ± 0.14	19.5 ± 1.3	23.4 ± 1.7	760 ± 46	3.20 ± 0.56	
HEI	L-CI	0.32 ± 0.05	11.4 ± 0.8	23.1 ± 1.5	278 ± 18	0.81 ± 0.15	1988–1990
	M-CI	0.49 ± 0.08	11.9 ± 1.1	18.1 ± 1.3	615 ± 28	2.26 ± 0.41	
	H-CI	0.75 ± 0.13	12.7 ± 1.3	16.3 ± 0.9	825 ± 41	3.57 ± 1.19	
ZGT	L-CI	0.29 ± 0.06	9.5 ± 0.7	26.8 ± 1.6	265 ± 21	0.80 ± 0.16	1985–1989
	M-CI	0.42 ± 0.07	9.3 ± 0.8	20.1 ± 1.4	635 ± 32	2.75 ± 0.34	
	H-CI	0.82 ± 0.14	9.9 ± 1.1	16.9 ± 1.2	1,025 ± 76	4.18 ± 0.90	
FUJ	L-CI	0.21 ± 0.05	12.7 ± 0.6	38.6 ± 2.1	210 ± 16	0.63 ± 0.40	1971–1973
	M-CI	0.38 ± 0.09	12.8 ± 0.7	33.8 ± 1.8	355 ± 22	1.73 ± 0.22	
	H-CI	0.72 ± 016	14.4 ± 1.2	21.1 ± 1.7	780 ± 40	3.21 ± 0.54	
HLB	L-CI	0.33 ± 0.07	16.8 ± 1.4	31.1 ± 1.7	275 ± 19	1.09 ± 0.21	
	M-CI	0.52 ± 0.11	17.5 ± 1.8	26.5 ± 1.8	550 ± 45	2.22 ± 0.17	
	H-CI	0.75 ± 0.13	18.2 ± 1.5	21.1 ± 1.4	950 ± 62	3.49 ± 0.41	

*Sites are Mu Us (MUS, Shanxi Province), Saihanba (SHB, Hebei Province), Heishui, Zhangutai, and Fujia (HEI, ZGT, FUJ: all Liaoning Province)*.

### Tree-Ring Width and Chronology

Core samples were collected (two samples per tree) at breast height (1.3 m) from 20 pines randomly selected from each plot. The samples were fixed and dried in the laboratory, polished with sandpaper, and analyzed for tree-ring width (TW) (precision: 0.01 mm; Lintab-6, Rinntech, Heidelberg, Germany). The results were cross-dated and verified with COFECHA software to eliminate measurement errors (Holmes, 1983). Then, data were detrended by regional curve standardization (RCS) with ARSTAN 4.4 software to generate residual chronologies. In this study, we aimed to investigate the growth-climate-competition relationships of the trees at the same stage in their life span. Core samples of the same time span were used for growth comparison at the same site. For each plot, 9–12 core samples meeting such criterion were selected for calculation of mean BAI.

### Competition Index

The distance-dependent CI was calculated for each subject tree (Hegyi, 1974) to characterize the intensity of competition in a stand:


(1) $$CI=\sum_{j=1}^{n}\left(\frac{D_{j}}{D_{i}}\cdot \frac{1}{d_{ij}}\right)$$

where D_j_ is the DBH of a competitor within 8 m; D_i_ is the DBH of the subject tree; d_ij_ is the distance between the subject tree and the competitor; and *n* is the number of competitors. The stand levels of CIs were categorized (Cescatti and Piutti, 1998) as low (CI < 1.0), medium (1.0 ≤ CI < 3), or high (CI ≥ 3).

### Reconstructing Annual CI

As competition varies during growth, annually recording the DBH of a subject tree would accurately depict the trend of competition. However, this was not feasible for the stands studied. Therefore, a method about the annual series of tree growth rate was used to reconstruct the annual increment data for all the trees in each plot (Calama et al., 2019). The annual series of tree ring growth was transformed into annual series of tree growth rate by dividing the observed radial increment in year k among total radial increment. Different trees were cored in each plot, and radial increment for each year was measured and averaged per tree. Growth data in year k within two inventories were computed for each cored tree as follows:


(2) $$GR_{k}=\frac{RI_{k}}{RI_{t}}$$

where GR_k_ is the growth rate in year k, RI_k_ is the radial increment in year k, and RI_t_ is the radial increment of the total year. The mean plot growth rate was computed by averaging the synchrony growth rate of cored trees in the same plot.

The diameter increment for a non-cored tree in year k was obtained by multiplying the total diameter of this tree by the plot growth rate for that year. The past size of the cored trees can be reconstructed by subtracting radial increments, while the past conditions of the non-cored trees are approached by fitting relation between the annual series of diameter increment and current diameter at breast height. We assumed that (i) the rate of annual diameter increment over bark is equivalent to the rate of annual radial increment under bark, and (ii) Mongolian pines of plantations in the same plot are of the same age because of the plantation, and no trees die during growth. The CI in year k was calculated with the following Equation:


(3) $$CI_{k}=\sum_{nc=1}^{n}\left(\frac{D_{nc-k}}{D_{c-k}}\cdot \frac{1}{d_{c-nc}}\right)$$

where CI_k_ is the competition index in year k, D_nc−k_ is the DBH in year k of a competitor (non-cored tree) within 8 m; D_c−k_ is the DBH in year k of the cored tree; d_c−nc_ is the distance between the cored tree and the competitor; and *n* is the number of competitors.

### Relative Impacts of Climate, Competition, and Site Condition on Radial Growth

The impacts of competition and climate on radial growth of different stand densities were differentiated by modeling. As the climatic, CI, and site index factors were temporally inconsistent, 5-year averages of the most significant climatic factors (Pearson's correlation; see section Data analysis) were calculated and used for modeling (Liang et al., 2019). The CI for the subject tree was obtained by reconstructing its BAI with Calama's model (Calama et al., 2019). The mixed model only contained climate, competition, and site index, while age was not considered because it is an inherent factor and followed a highly significant logarithmic relationship with Log BAI (Figure S2). Then, using the R package “relaimpo,” the climatic factors and modeled CI were used to differentiate the impacts of climate, competition, and site index during the growth period (Grömping, 2006; Liang et al., 2019).

### Vulnerability to Drought

Drought condition can be classified using the SPEI. In this study, the SPEI for each year was calculated for the growing season spanning seven months from April to October. When SPEI of the growing season was under the value of −1, the year was considered a severe drought year (Merlin et al., 2015). Mongolian pine had an obvious decrease in growth during the severe drought spell. Several previous research studies found that drought legacy effects usually lasted 1–2 years (Castagneri et al., 2018; Huang et al., 2018). We used a criterion that the two drought events had to be temporally separated by more than 3 years to avoid counting multi-year single droughts as two different droughts based on the previous study (Anderegg et al., 2020). Following this criterion, three drought events were selected for MUS (1996, 2008, and 2014), SHB (1985, 2008, and 2014), ZGT (2002, 2008, and 2014), FUJ (1985, 1993, and 2008), and HLB (1985, 2003, and 2008), while two drought events for HEI (2008 and 2014). Four indicators of tree response to severe drought events were calculated: resistance (Rt), recovery (Rc), resilience (Rs), and relative resilience (RRs) with a 3-year moving average BAI (Lloret et al., 2011).


(4) $$\text{Resistance Rt }=\;{\text{BAI}}_{\text{D}}{\text{/BAI}}_{\text{pre}}$$

(5) $$\text{Recovery Rc }=\;{\text{BAI}}_{\text{post}}{\text{/BAI}}_{\text{D}}$$

(6) $$\text{Resilience Rs }=\;{\text{BAI}}_{\text{post}}{\text{/BAI}}_{\text{pre}}$$

(7) $$\text{Relative resilience RRs }=\;({\text{BAI}}_{\text{post}}-{\text{BAI}}_{\text{D}}){\text{/BAI}}_{\text{pre}}$$

where BAI_D_ is the mean BAI during a drought event, and BAI_pre_ and BAI_post_ are the mean BAIs in the 3-year span before or after the event.

### Meteorological Data, SPEI, and VPD

Meteorological data for five of the forests were provided by nearby national standard meteorological stations (MUS: station No. 53646; HEI: station No. 54324; ZGT: station No. 54236, FUJ: station No. 54142; HLB: station No. 50527). Data for SHB were obtained from the local meteorological station of the plantation. To ensure meteorological data (e.g., temperature, precipitation, relative humidity, wind speed, and atmospheric pressure) were representative of the local climatic trend, they were checked with Mann–Kendall and double-mass tests for homogeneity to confirm the data were free from random variations. The SPEIs were calculated at intervals of 1 and 12 months with the SPEI program (Vicente-Serrano et al., 2010) to reveal relatively small changes in drought conditions on monthly and yearly scales, respectively. The VPD of the air was calculated from temperature and relative air humidity.

### Data Analysis

Tree growth is affected by the climatic factors of the current period and the preceding periods (Sánchez-Salguero et al., 2015a; Liang et al., 2019). Therefore, the Pearson correlations of climate-growth were analyzed with the R package “treeclim” using meteorological data spanning the previous October to the current September (Zang and Biondi, 2015). Then, the most significant climatic factors were identified according to the Pearson correlation coefficients and used for calculating the relative contributions of climate, competition, and site index on growth (Section 2.5). The TW, BAI, Rt, Rc, and Rs of the different densities trees were analyzed with the least significant difference (LSD) test (SPSS 20, IBM, Armonk, NY, USA), and *p* < 0.05 was considered statistically significant.

## Results and Analysis

### Meteorological Factors

**Figure 2:**
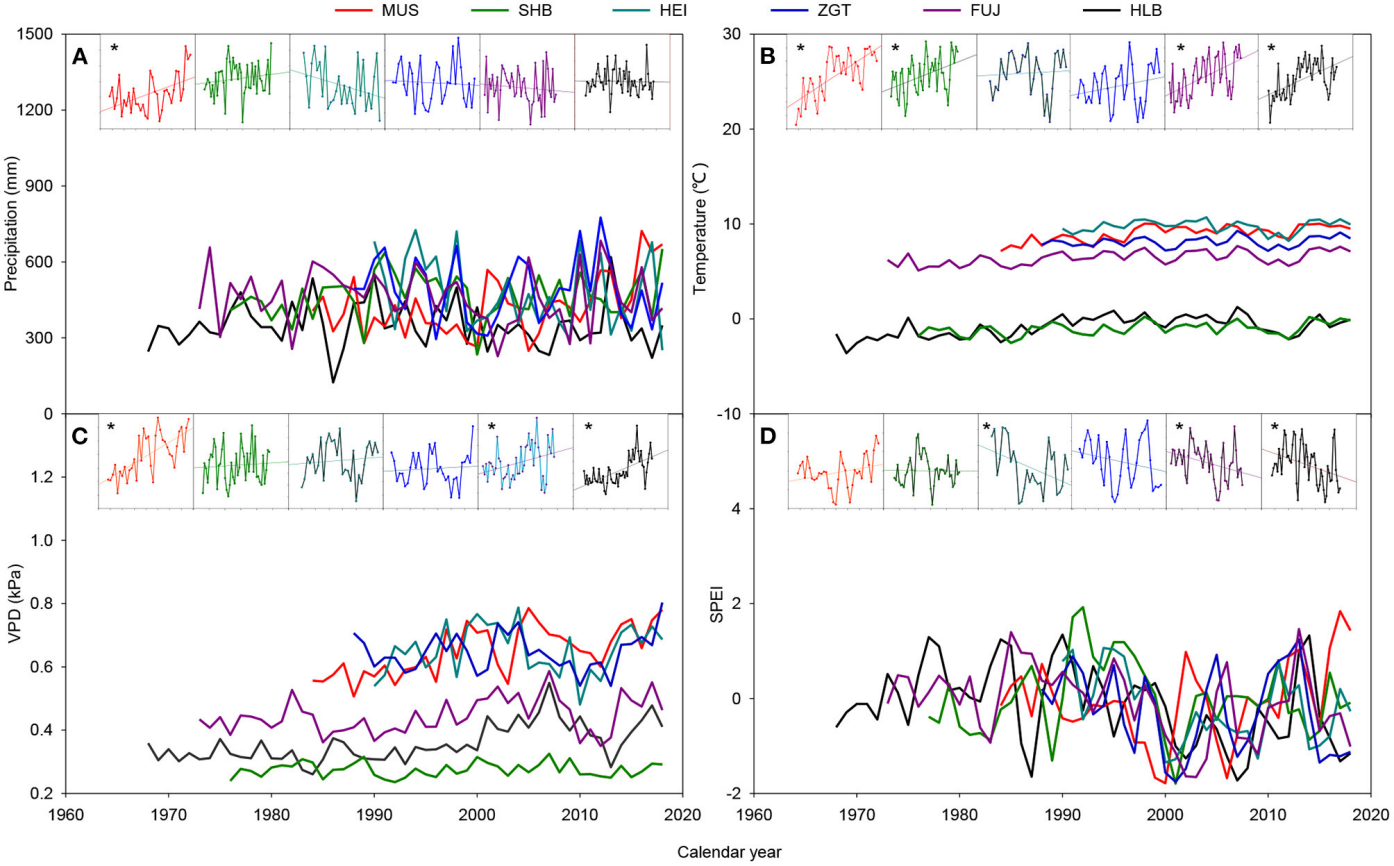
Variation in total precipitation **(A)**, mean temperature **(B)**, mean vapor pressure deficit (VPD) **(C)**, and mean standardized precipitation evapotranspiration index (SPEI) **(D)** during the growth period at six experimental sites in the Three-North Shelter Forest. Asterisks indicate that the trend was a significant increase or decrease at *p* < 0.05.

The MUS and SHB sites displayed trends of increasing annual precipitation with the year of study, whereas HEI, ZGT, and FUJ had the opposite trend (Figure 2). The site at HLB remained relatively stable. All sites showed trends of warming, with significant increases for SHB, MUS, FUJ, and HLB (*p* < 0.05). All sites had elevated VPD with time, with the trend being particularly evident for MUS, FUJ, and HLB (*p* < 0.05). The SPEIs of HEI, FUJ, and HLB tended to have a significant decrease with the year of study (*p* < 0.05), whereas MUS had the opposite trend. The site at SHB and ZGT showed a slight decrease. Overall, during the period studied, MUS has become warmer and more humid, while HEI, ZGT, FUJ, and HLB have become warmer and more arid. In contrast, SHB had shown increasing annual precipitation and temperature without a marked change in SPEI.

### Radial Growth Analysis

**Figure 3:**
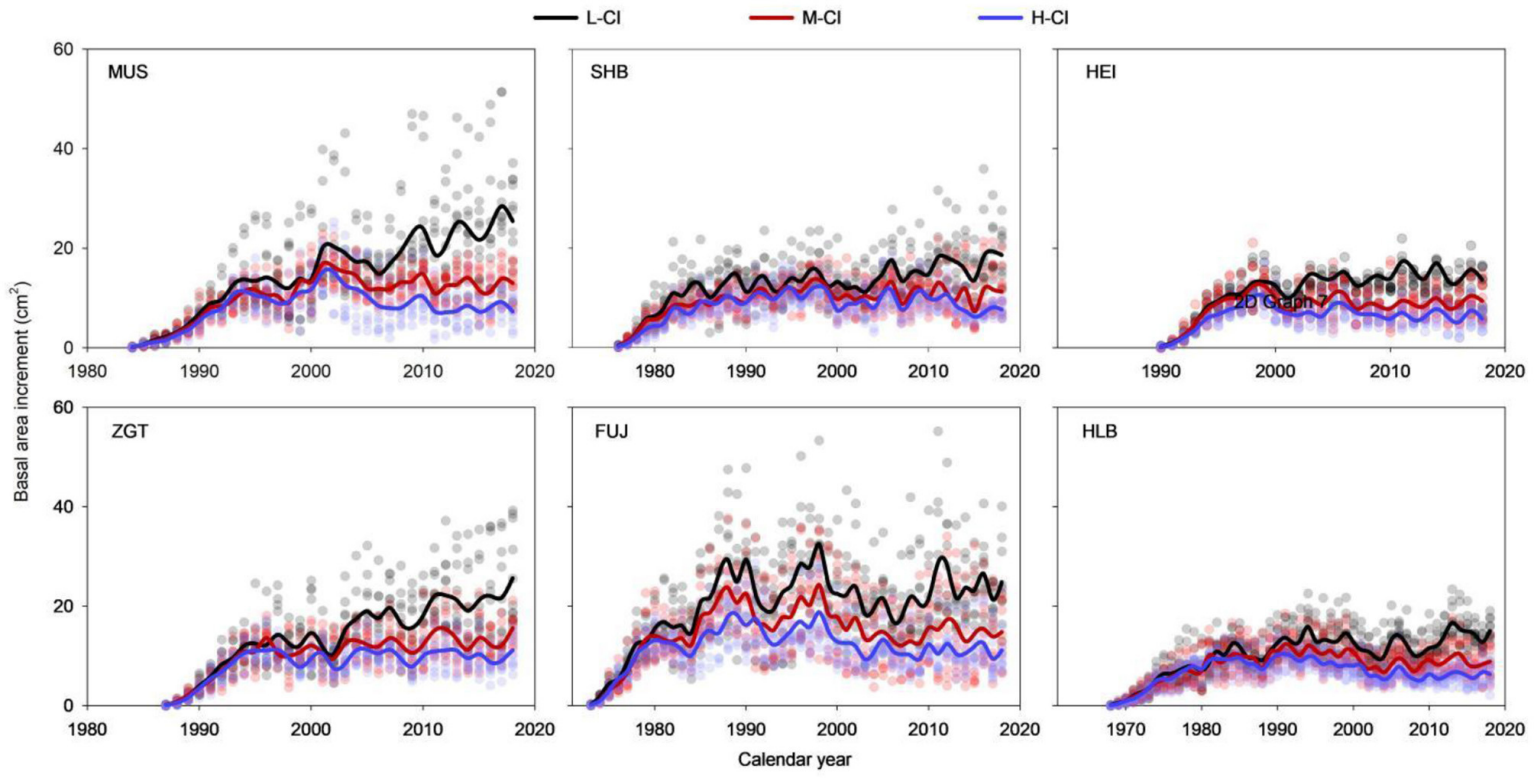
Differences in basal area increments (BAIs) among Mongolian pine trees at the six experimental sites.

The BAIs measured from all stands and sites exhibited a steady increase during the first 10 years followed by substantial fluctuations (Figure 3). The low- and high-CI stands trended down and up, respectively, while medium-CI stands remained relatively stable. In the later stage of the life span, the high- and medium-CI stands had evidently lower BAIs than low-CI stands. Statistical analyses found that, for SHB, the BAIs measured from the stands of different CIs were not significantly different in the first 5 years (Figure S3); subsequently, the BAIs from the low-CI stands were significantly higher than those from high- and medium-CI stands (all *p* < 0.05). Similar trends were observed for HLB (i.e., no significant difference only in the first 15 years) and MUS, HEI, ZGT, and FUJ (i.e., no significant difference only in the first 10 years).

### Relationship Between Radial Growth and Environmental Factors

**Figure 4:**
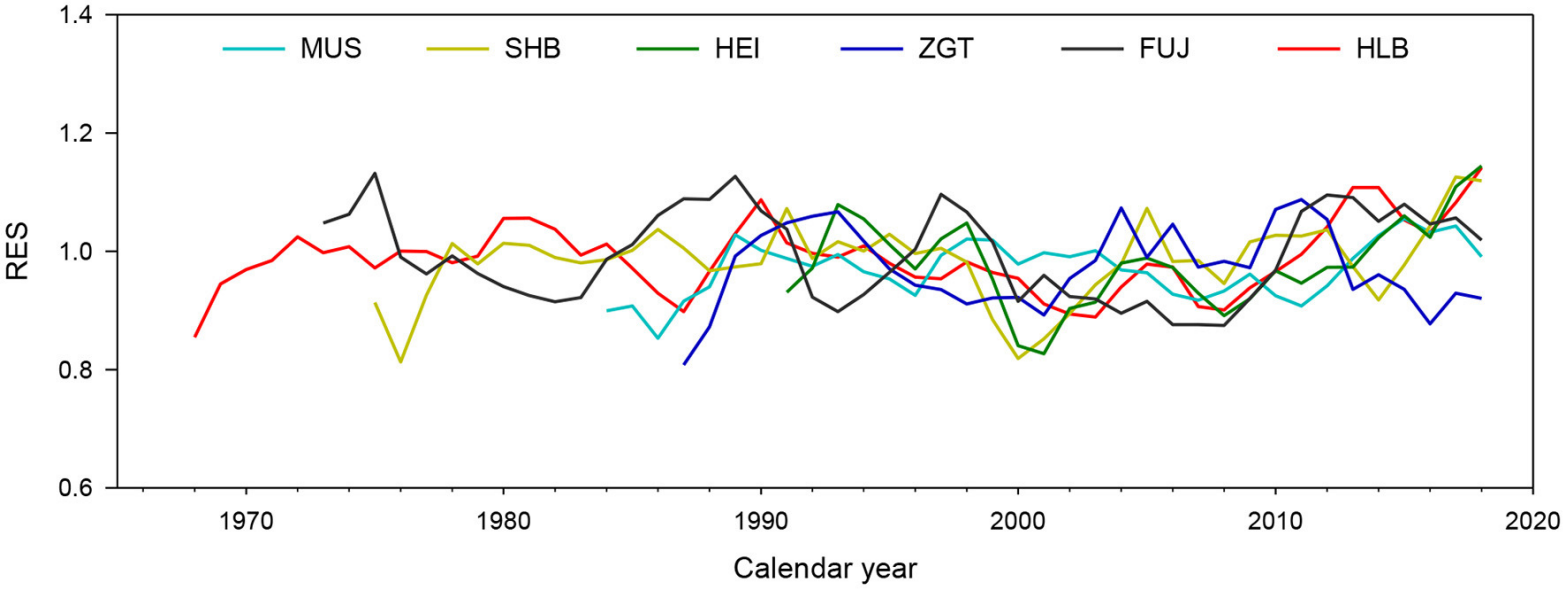
Individual time-series and residual tree-ring width (TW) index among the Mongolian pine trees at six experimental sites.

Residual chronologies for the six experimental sites did not vary consistently, most likely because of their different habitats (Figure 4). Calculations gave a mean correlation coefficient (Rbar) of 0.142–0.225 and an expressed population signal (EPS) of 0.945–0.973, indicating that the signal from the samples effectively represented the overall characteristics of their populations. All signal-noise ratios (SNRs) were found to be >17.14, indicating that the chronologies represented high-frequency climatic variations and were thus suitable for dendroclimatological analysis (Table S2).

**Figure 5:**
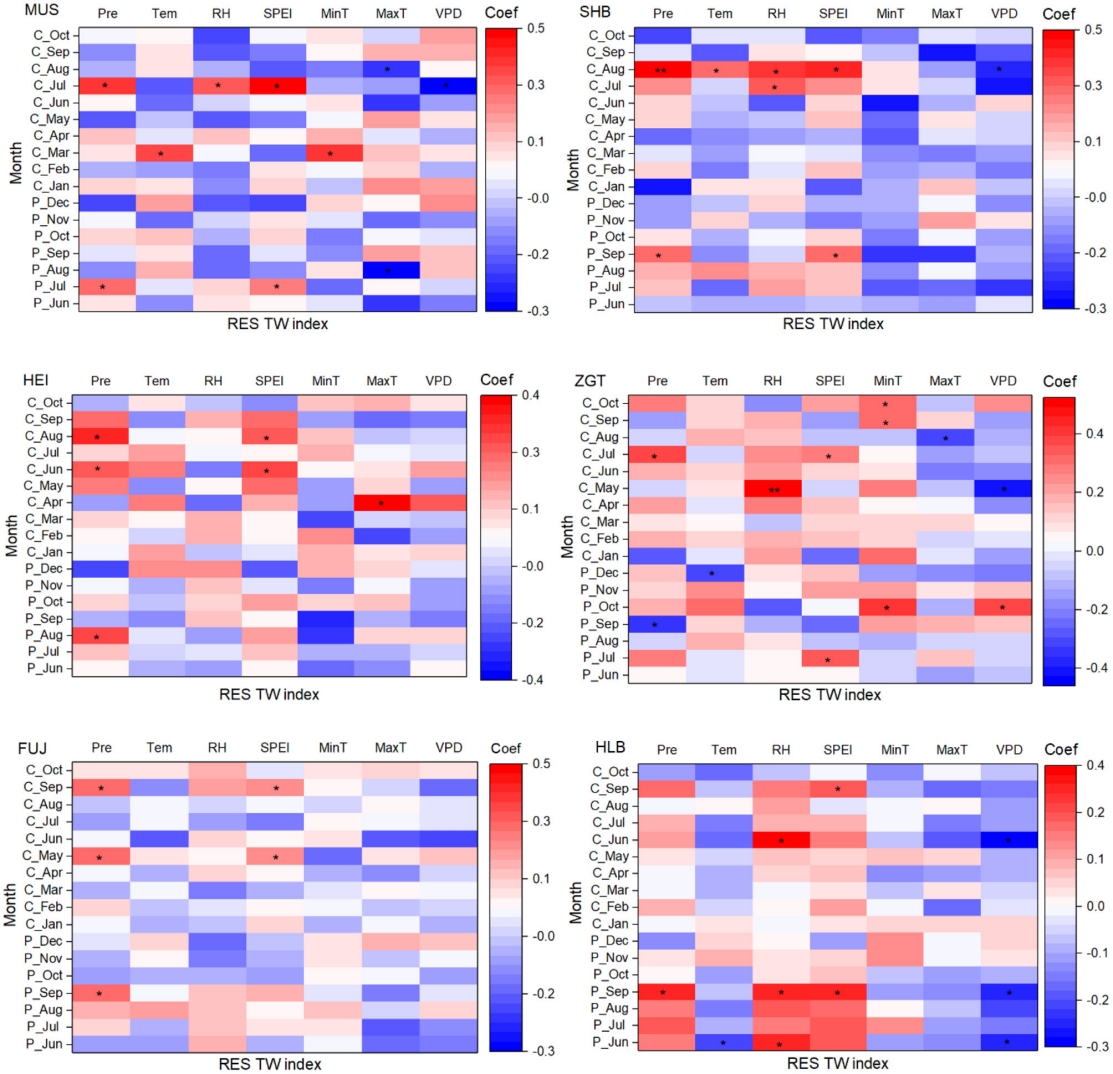
Relationships between the residual tree-ring width (RES) index and monthly climatic factors for all of the sites at a monthly timescale. C_month represents the current year of ring formation, and P_month represents the climate variables during the year preceding ring formation. *p < 0.05 and **p < 0.01, respectively.

In North China, the growing season of Mongolian pines spans April–October. The association between their growth and climatic factors was determined by Pearson correlation (Figure 5). For MUS, the residual TW index was closely related to environmental factors in July, including significant positive correlations with precipitation, RH, and SPEI in the July of the current year and precipitation and SPEI in the previous July, and a significant negative correlation with VPD of the current July (all *p* < 0.05). For SHB, the index was closely related to environmental factors of the current August, including significant positive correlations with precipitation (*p* < 0.01), temperature and RH (both *p* < 0.05), and a significant negative correlation with VPD (*p* < 0.05). For HEI, the residual TW index was closely related to environmental factors of June and August, including significant correlations with the SPEIs of the current June and August, as well as precipitation in the previous August (all *p* < 0.05). For ZGT, the index was significantly positively correlated with five factors [RH of the current May (*p* < 0.01) and precipitation and SPEI of the current July (both *p* < 0.05), and temperature nadirs recorded in the current September and October (*p* < 0.05)] and was significantly negatively correlated with the VPD of current May (*p* < 0.05). For FUJ, the residual TW index was significantly positively related to the precipitation and SPEI in both the current May and September (all *p* < 0.05). For HLB, the index was significantly positively related to the RH of the current June and significantly negatively related to the current June VPD; it was also significantly positively correlated to the precipitation, RH, and SPEI of the last September and significantly negatively correlated to the VPD of that month (*p* < 0.05). Overall, these showed an acceleration of growth from increased precipitation, RH, and SPEI, and inhibition of growth from increasing VPD and maximum temperature.

### Relative Contribution of Climate, Competition, and Site Condition on Mongolian Pine Growth

**Figure 6:**
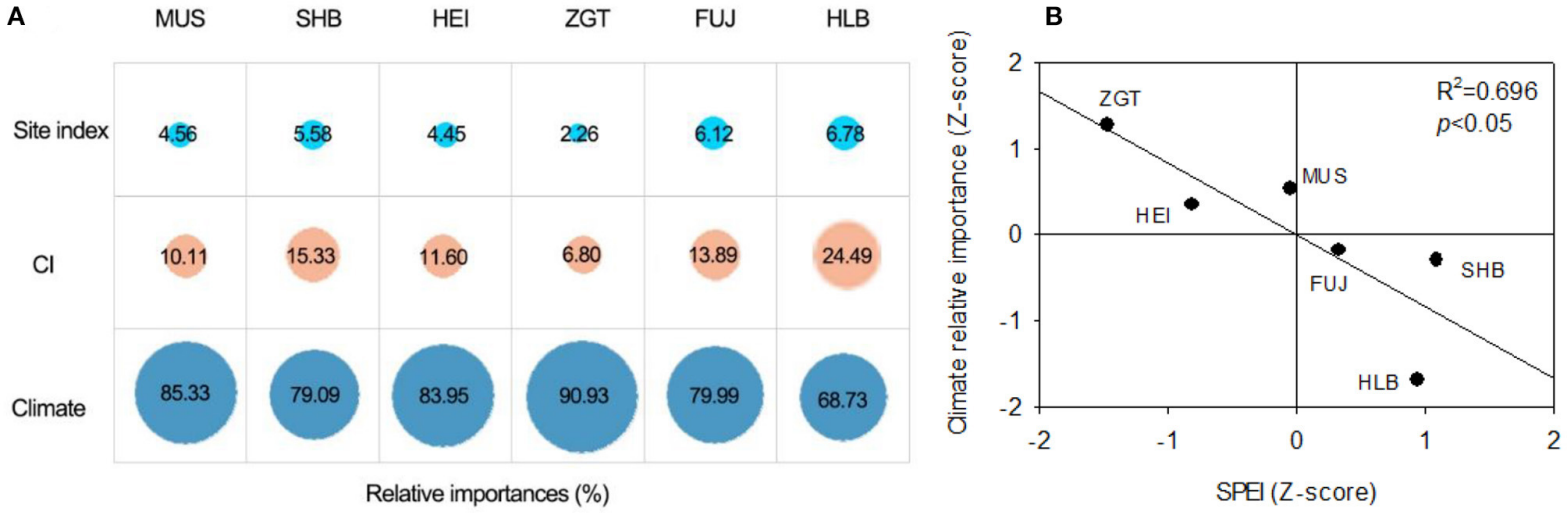
**(A)** Relative importance of climate and competition on tree radial growth of Mongolian pines at six experimental sites. **(B)** The climate relative importance is significantly correlated with the SPEI at different experimental sites (*p* < 0.05).

It was found that the growth of Mongolian pines at all sites was mainly affected by climatic factors, while the competition was the second and the site index was the smallest. For natural forest at HBL, the relative contribution of the climatic factor was the least (68.73%) among all sites (Figure 6A). For the plantation forests at the other sites, the contributions of climatic factor increased. Linear correlation analysis revealed that the relative impact of climate increased with the difference in SPEI (plantation sites vs. HBL), showing a significant correlation (*p* < 0.05; Figure 6B), while the reverse pattern of the impact of competition was observed.

### Effects of Competition on Vulnerability to Drought

**Figure 7:**
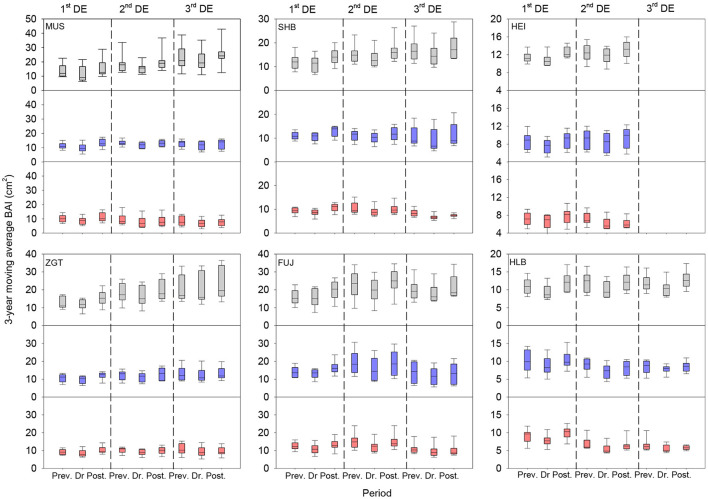
Three-year moving average BAIs for the six Mongolian pine sites before, during, and after drought events. The boxplots show the mean values for all sites during the two or three drought events. DE represents drought event. Gray, blue, and red boxes represent the low-CI, medium-CI, and high-CI of Mongolian pine trees, respectively. CI, competition index.

The three-year moving average BAI revealed that all six sites were affected by up to three drought events, and stands showed different patterns of growth before and after drought regardless of the level of CI (Figure 7). All stands displayed decreased BAIs during drought (vs. pre-drought BAI) regardless of CI level. For all drought events, the low-CI stands recovered or even exceeded the pre-drought BAIs when the event terminated. The medium-CI stands recovered, but did not exceed, the pre-drought BAIs after each event. In contrast, the high-CI stands recovered only after the first event, with lower BAIs (vs. pre- or during-drought levels) after the second or third event.

**Figure 8:**
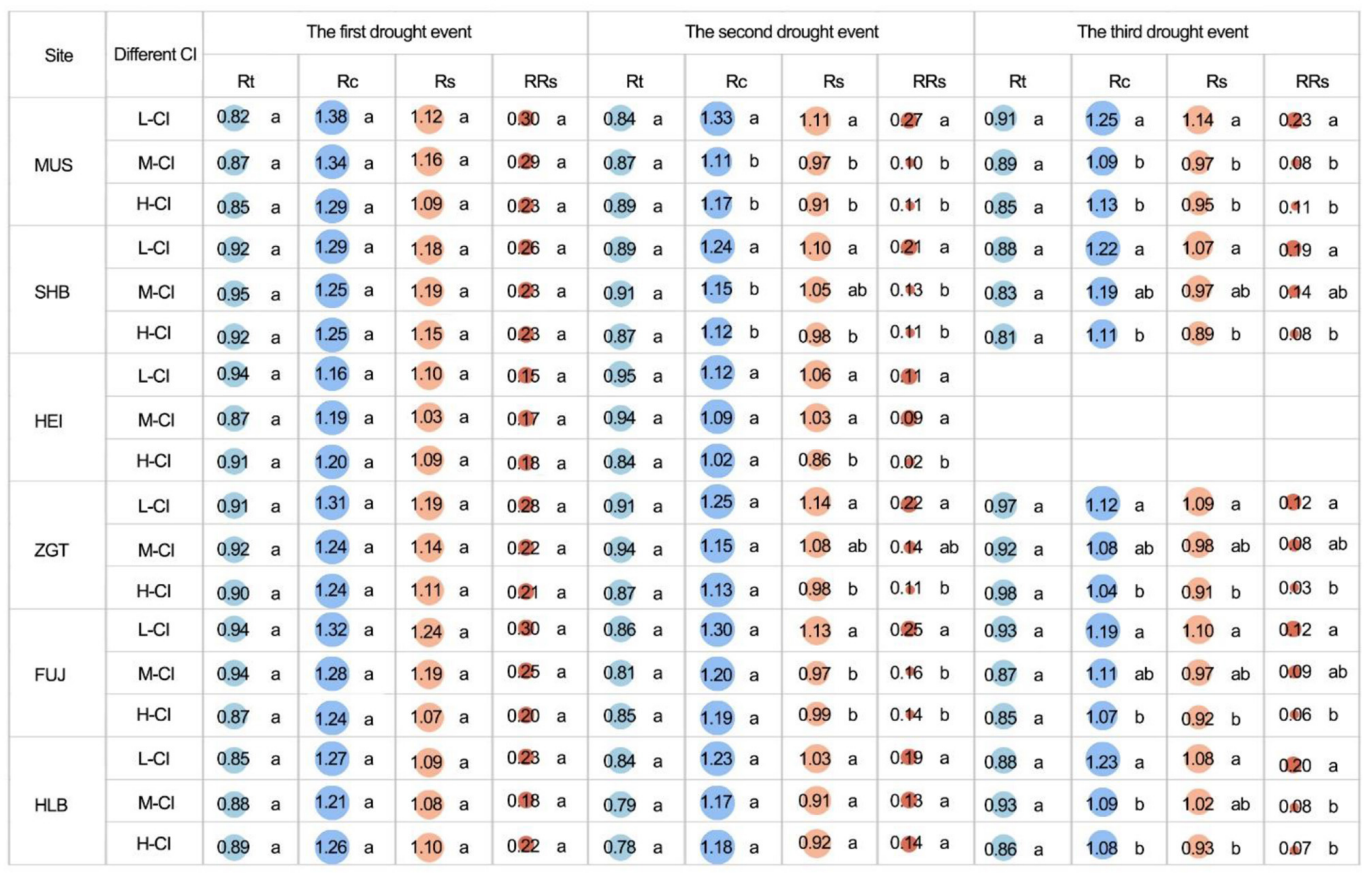
Resistance (Rt), recovery (Rc), resilience (Rs), and relative resilience (RRs) among the all competition index (CI) groups of Mongolian pine trees. Lower-case letters indicate significant differences at *p* < 0.05.

The Rt recorded from stands of different CIs was not significantly different (*p* > 0.05) despite the experience of multiple drought events (Figure 8), suggesting that Rt may be related to the species characteristics. During the first event, the Rc from stands of different CIs was not significantly different (*p* > 0.05). However, in the second event, for MUS and SHB, the CIs recorded from medium- and high-CI stands were significantly different (all *p* < 0.05). In the third event, for all sites except HEI, the CIs recorded from high-CI stands were at least 7.14% lower than those from low-CI stands, and the differences were statistically significant (all *p* < 0.05). Similarly, regardless of site, Rs from stands of different CIs was not significantly different in the first event. In the second event, however, for all sites except HLB, the high-CI stands had significantly lower Rs compared with the low-CI ones. In the third event, significantly lower Rs was also observed in high-CI stands (vs. low-CI ones) for all sites except HEI, which did not undergo the third drought. From the second event, the RRs of the high-CI stands were only 25–50% of the low-CI stands, indicating more severe damage from drought in high-density stands, compared to low-density stands (Figure 8).

**Figure 9:**
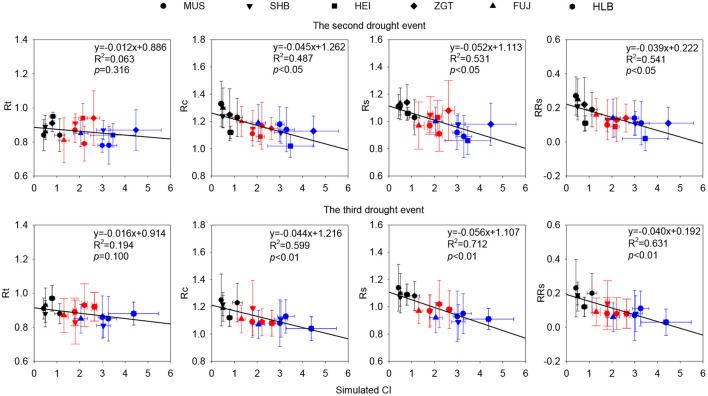
Correlations of simulated competition indices (CIs) with Rc, Rs, and RRs (*p* < 0.05) at all densities during the second and the third drought event. Simulated CI was reconstructed with a model of annual series of tree growth rate (Calama et al., 2019). Black, red, and blue symbols represented L-CI, M-CI, and H-CI, respectively.

Correlation analysis found that, in the first drought event, Cl was not significantly correlated with Rt, Rc, Rs, or RRs regardless of all densities (Figure S4). In the second event, however, for all densities, CI was significantly negatively correlated with Rc, Rs, and RRs (all *p* < 0.05). In the third event, CI was significantly negatively correlated with Rc, Rs, and RRs (*p* < 0.01). The overall decrease in the three parameters with CI suggests their density-dependent nature (Figure 9).

## Discussion

### Effects of Climate, Competition, and Site Condition on Growth

Drought and high temperature can all reduce the growth of trees (Sánchez-Salguero et al., 2015a; Foster et al., 2016; Anderegg et al., 2020). Severe stress creates physiological damage in trees; as a result, they adopt defensive strategies such as defoliation and a reduction in vigor, thus displaying slower growth and declining stand quality (Greenwood et al., 2017). Water and temperature have been established as key factors controlling tree growth. This study found that, for all six sites, the TW indices were positively correlated with precipitation, RH, and SPEI, and negatively correlated with VPD and peak temperature. The impact of these factors, however, differed between sites, most likely mainly because of the different climatic factors. The growth of the natural forest in HLB was closely related to the climatic factors of the previous year (RH, SPEI, VPD, and precipitation last September). In comparison, the growth of the plantation forests in the other five sites was closely related to climatic factors of the growing season of the current year, particularly precipitation and SPEI. The carbon assimilated by photosynthesis of natural forest in high latitude area was not depleted in the current year and would be used to build the new tissues in the next year, while the wood formation of Mongolian pine in low latitude area used more carbohydrates of the current year (Zeng et al., 2019). A recent study found that the TW index of Mongolian pines in Horqin Sandy Land (Inner Mongolia, China) was significantly positively correlated with the total precipitation in May–July of the current year and significantly negatively correlated with the mean temperature of the same period (Li et al., 2020). The growth of *Pinus sylvestris* L was dependent on precipitation in April–July of the current year, while there was an impact of moisture deficit during May of the current year on annual height growth of Scots pines (Taeger et al., 2013). Water availability negatively affects tree growth (Foster et al., 2016; Sohn et al., 2016). Reduced SPEI (Gomes Marques et al., 2018) and increased VPD in summer (Trugman et al., 2018) have been observed to reduce forest productivity. However, the response of a tree to temperature and water stress may vary. Foster et al. (2016) studied 15 tree species and found that the effects of temperature and summer water stress interacted, such that warm years produced a positive response to summer water availability, whereas cold years produced a negative response. A previous study concluded from Scots pines growing under continental Mediterranean climates that, at a regional scale, the balance between the positive and negative effects of summer precipitation and winter temperature on radial growth is both elevation- and latitude-dependent, with those in low elevations more vulnerable to drought and heat stress (Sánchez-Salguero et al., 2015a) but those at high altitudes not (Shi et al., 2019).

Competition exists primarily between neighboring trees, fundamentally for resources such as sunlight, water, and nutrients. It has an evident impact on tree growth. In all six sites, the low-CI stands had greater DBHs than medium- or high-CI stands. During the early stage of growth (5–15 years), the stands of different densities showed no significant difference in TW or BAI. In the subsequent stage, however, the growth of medium- or high-CI stands decelerated and became significantly lower than low-CI ones over several decades, indicating a competition-restrained growth (Figure S3). Other studies observed that high competition negatively affected tree growth as BAI and CI followed negative exponential functions (Sánchez-Salguero et al., 2015b). Moreover, the responsiveness to climate increased with decreasing competition, indicating a loss of growth sensitivity to climate with increasing competition. Maintaining forests at lower density might enhance resilience to drought in all climates, but the interactions between drought and competition on forest growth were difficult to tease apart. Drought was the dominant control on forest growth in the more arid sites, whereas density, drought, and their interactions were controlling factors in the more humid sites (Gleason et al., 2017). The interactions between climatic factors and competition at all sites were not significant in this study because the Mongolian pine is adaptable in such an arid area.

Except for climate and competition, the growth of the tree is also controlled by size, age, and site condition that may produce different effects. Previous studies concluded that tree growth in some regions or at one site is predominantly controlled by competition (Zhang et al., 2015; Liang et al., 2019), with the impact of climate being secondary (Gómez-Aparicio et al., 2011). This trend was considered particularly evident for dominant species (Jiang et al., 2018). However, few studies focused on quantifying the effects of climate and competition and site condition during the growth period. Quantitative analyses in this study indicated that, for all six sites, climatic factor contributed >68.73% of the overall impact on growth, substantially overriding the most significant competition factors. In mature forest, long-term competition leads to declining health and regeneration, and greater mortality, of trees, acting as a driver of compositional changes of mixed forest in North China (Zhang et al., 2015). However, the competition increased with the growth in the young stage, which leads to the relative impact of competition lower than that of climate in immature Mongolian pine plantation. Notably, in our study, for the natural Mongolian pine stands in its native range (HLB), the effect size of competition on growth was 24.49%. In comparison, for trees distributed in plantation forests (i.e., outside native range), the relative impact of competition decreased but that of climate increased (Figure 6A). Liang et al. (2019) reported that, on a large spatial scale, the relative impact of competition on the radial growth of subtropical trees decreased from Southeast China to North China, whereas the impact of climate showed the opposite trend. The species is another variable that shifts markedly along the climatic gradient (Gómez-Aparicio et al., 2011). Although climate-mediated species migration is not evident in analyses at continental scales, forests may undergo faster turnover in adaptation to climate changes (Zhu et al., 2014). Site condition is one of the influencing factors of growth. In our study, the site conditions have little effect on its growth due to Mongolian pines at six sites were planted in the sandy soil of the plain. With increasing frequencies of drought and high-temperature events, competition between trees for local resources inevitably escalates. As a result, the impact of competition on growth frequently increases at sites with poor conditions (Calama et al., 2019).

### Divergent Vulnerability to Repeated Drought

Severe drought leads to tree decline and mortality, compromising the health and ecosystem services of forests (Trumbore et al., 2015). Loss of forest productivity is related to thresholds of specific components of its ability to recover growth. As the majority of trees experience multiple drought events in their lifetime, post-drought recovery is critical to their long-term survival. In this study, the stands of different CIs did not differ significantly in Rt but did so in Rc, with the high-CI stands having significantly lower Rc than low-CI stands. This indicates a reduced ability of the high-density stands to recover pre-drought growth following drought. Although the drought tolerance of trees was significantly negatively related to the severity of drought, the association between post-drought recovery and drought severity was weak (Wang et al., 2020). Instead, drought resistance and recovery may be linked to multiple factors such as carbon reserves, stand density, and age. Galiano et al. (2011) proposed that, if the resistance and recovery were both partly determined by energy reserves, a trade-off between post-drought resistance and recovery was inevitable. The low Rc of the high-CI stands may be attributable to their low carbon reserves and low radial growth. Other studies observed that reducing stand density improved drought resistance (Manrique-Alba et al., 2020) and recovery (Sohn et al., 2016) by mitigating competition for resources and improving soil water availability. Young trees were reported to have better drought resistance than adult ones (Andivia et al., 2020), consistent with the lack of significant difference between Rt and Rc of stands of different densities observed here. Taken together, the findings of this study and earlier studies all indicate that tree response to drought is a complex process (Lloret et al., 2011; Pardos et al., 2021).

The ability of trees to regain their pre-drought growth rate after the drought is characterized by Rs, with Rs < 1 indicating a sustained negative impact of the drought on subsequent growth. If the growth rate fails to return to the pre-drought level, RRs approach 0 and can even become negative. With the exception of the first drought event, we observed an evident decrease of Rs in high-CI stands. This indicates an adverse impact of the drought event on the high-density stands, which prevented them from fully regaining pre-drought growth rates. After the second (except for HLB) and third (except for HEI) drought events, the high-CI stands exhibited significantly lower RRs than the low-CI ones (all *p* < 0.05). Stresses and damages from multiple drought events accumulated, leading to reduced vigor, tree declines, and mortality. For HLB, the difference became statistically significant only at the third event. This suggests a higher resilience in natural forests (vs. plantation ones), consistent with the greater resistance and recovery of natural (vs. plantation) pine forests reported by Rubio-Cuadrado et al. (2018). Other studies found that, in Mongolian pine forests of increasing ages, the crown radius enlarged with stand density, generating increasing crown overlap (Sun et al., 2020). Competition is a determining factor of tree growth, with high-density stands more likely to develop decelerated growth and declining vigor. A negative association between population density and recovery indicates that trees in low-density stands were less affected by drought events. Chauvin et al. (2019) compared Douglas firs of different provenances and found that those from more arid regions had higher resistance to cavitation and drought. Scot pines of different provenances differed moderately in resistance to drought but had significant differences in Rs and RRs, highlighting the importance of provenance in investigating tree vulnerability under a changing climate (Taeger et al., 2013). Reduced growth and increased mortality may be related to thresholds of specific components of resilience rather than to a total loss of resilience over time (Lloret et al., 2011). In our study, the high-CI stands did not lose resilience completely (Figure 8), consistent with this suggestion. However, for many sites, the RRs of high-CI stands decreased stepwise to approach 0, indicating a cumulating impact of drought on these stands. DeSoto et al. (2020) investigated the drought tolerance of a number of species and concluded that drought-related mortality of angiosperms is associated with lower resistance. The results of this study agree with their findings. Heavy thinning improved the drought resistance and resilience of Mediterranean pines, whereas densely planted trees were vulnerable to a warmer and drier climate (Navarro-Cerrillo et al., 2019). Although thinning could alleviate growth declines during drought, the effects on growth after stress were uncertain (Castagneri et al., 2021).

Drought reduces the growth and biomass in forests of all ages (Anderson-Teixeira et al., 2013), but a high post-drought growth resilience is likely to be crucial for their long-term survival (DeSoto et al., 2020). In our study, Rc, Rs, and RRs decreased with increasing CI; in particular, for the second and third drought events, there was a significant negative correlation with density, exhibiting a density-dependent vulnerability to drought (Sun et al., 2020). For trees in high competition forests, the overall effect of climate and competition increased their vulnerability (Primicia et al., 2015), particularly during periods of high within-stand competition (after 40–50 years) (Panayotov et al., 2016). Tree response to drought is accompanied by legacy effects (Castagneri et al., 2018) that may create a negative impact for years (Huang et al., 2018). After surviving multiple drought events, the overall effects of drought and density changed the sensitivity of drought response. Recent studies predict that, in the future, high-CI Mongolian pine forests will be more susceptible to drought and more likely to develop hydraulic dysfunction (Li et al., 2020) and carbon starvation (Trifilò et al., 2017), thus facing greater risks of mortality. Maintaining forests at lower density is expected to improve the resource availability for trees (Sohn et al., 2016) and, thereby, improve their drought resistance, post-drought recovery, and adaptation to future severe conditions (Manrique-Alba et al., 2020). This suggests that, for arid North China, density-related competition should be considered in plantations of Mongolian pine forests and that thinned forests are more likely to adapt to future climate changes.

## Data Availability Statement

The original contributions presented in the study are included in the article/Supplementary Materials, further inquiries can be directed to the corresponding author/s.

## Author Contributions

SS, JZha, PM, and CY conceived the idea and contributed to the study design, discussed the results, and wrote the manuscript. JZho, CG, SL, and SS performed data collection in the field and contributed to chronology data analysis. SL and SS performed meteorological data collection and analysis. JZha, PM, and CY funded the study. All authors contributed to the interpretation of the results, discussion, and approved the final manuscript.

## Funding

This work was supported by the National Key Research and Development Project (Grant No. 2020YFA0608101), the National Non-profit Institute Research Grant of the Chinese Academy of Forestry (CAFYBB2018ZA001), and the Project of the Co-Innovation Center for Sustainable Forestry in Southern China of Nanjing Forestry University.

## Conflict of Interest

The authors declare that the research was conducted in the absence of any commercial or financial relationships that could be construed as a potential conflict of interest.

## Publisher's Note

All claims expressed in this article are solely those of the authors and do not necessarily represent those of their affiliated organizations, or those of the publisher, the editors and the reviewers. Any product that may be evaluated in this article, or claim that may be made by its manufacturer, is not guaranteed or endorsed by the publisher.
