# Supplementary material for: Long-Term Effects of Climate and Competition on Radial Growth, Recovery, and Resistance in Mongolian Pines
Source: Front Plant Sci. 2021 Sep 14;12:729935. doi: 10.3389/fpls.2021.729935 (PMC8477062; doi:10.3389/fpls.2021.729935)
Supplement: Supplementary file 1 [file Table_1.docx]

**Supplementary material**


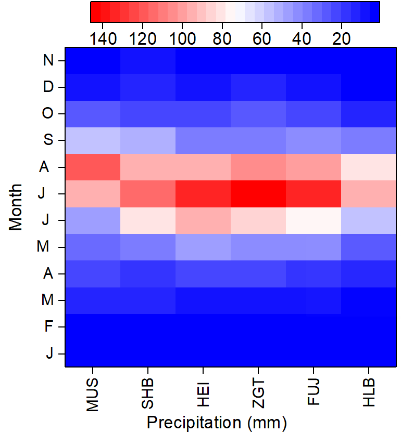

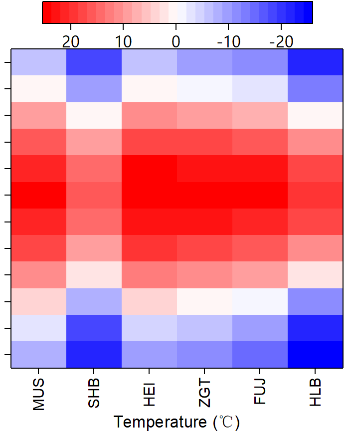

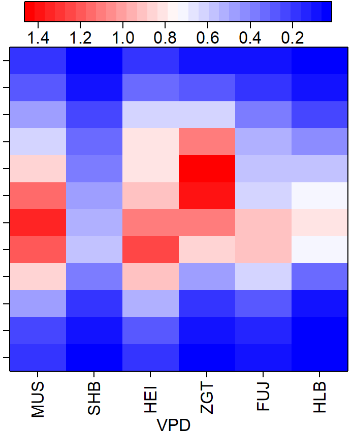

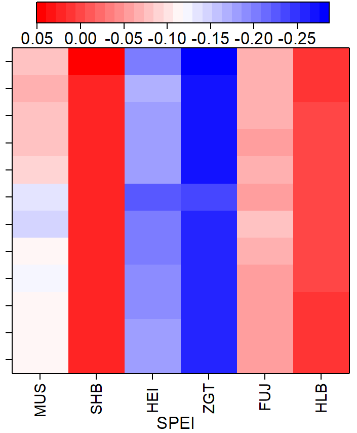


**Figure S1.** Variations in mean precipitation, temperature, vapor pressure deficit (VPD) and standardized precipitation evapotranspiration index (SPEI) at a monthly timescale.

**Figure S2.** For all of the sites, the Log BAI followed a highly significant logarithmic relationship with age (*p* < 0.001).

**Figure S3.** Differences in basal area increments (BAIs) for the all CI groups in different growth periods. Lower-case letters indicate significant differences at *p*<0.05.

**Figure S4.** Simulated CIs were no significantly correlated with resistance (Rt), recovery (Rc), resilience (Rs), and relative resilience (RRs) (*p*>0.05) during the first drought event. Black, red and blue symbols represented L-CI, M-CI and H-CI, respectively.

**Table S1**. Site location, elevation, annual average temperature, precipitation, VPD and SPEI during growth.

| site | Latitude and longitude | Elevation  (m) | Temperature (℃) | precipitation  (mm) | VPD | SPEI |
| --- | --- | --- | --- | --- | --- | --- |
| MUS | 109°19'E, 38°57'N | 1283 | 9.0 | 425 | 0.65 | -0.09 |
| SHB | 117°15'E, 42°24’N | 1553 | -1.0 | 465 | 0.28 | -0.01 |
| HEI | 119°26'E, 42°02'N | 525 | 9.7 | 476 | 0.65 | -0.19 |
| ZGT | 123°48'E, 43°20’N | 217 | 8.1 | 493 | 0.65 | -0.26 |
| FUJ | 123°49’E, 43°14’N | 127 | 6.4 | 462 | 0.45 | -0.07 |
| HLB | 119°28’E, 49°06’N | 603 | -0.9 | 350 | 0.36 | -0.02 |

**Table S2.** Characteristic value statistics of standard chronology. Trees/Cores, number of all cross-dated trees and cores; Rbar, mean correlation coefficients between cores; EPS, expressed population signal; SNR, signal-noise ratio; MS, mean Sensitivity.

| Site | Tree/Cores | Time span | Stand age | Rbar | EPS | SNR | MS |
| --- | --- | --- | --- | --- | --- | --- | --- |
| MUS | 61/122 | 1984-2018 | 27～35 | 0.146 | 0.945 | 17.141 | 0.098 |
| SHB | 71/142 | 1976-2018 | 36～43 | 0.190 | 0.973 | 36.454 | 0.123 |
| HEI | 58/116 | 1990-2018 | 24～28 | 0.225 | 0.961 | 24.389 | 0.139 |
| ZGT | 61/122 | 1987-2018 | 25～31 | 0.142 | 0.951 | 19.228 | 0.128 |
| FUJ | 57/114 | 1973-2018 | 39～46 | 0.240 | 0.965 | 27.403 | 0.110 |
| HLB | 69/138 | 1968-2018 | 40～50 | 0.182 | 0.969 | 30.793 | 0.099 |
